# 1,2-Bis(1,3-dithiol-2-yl­idene)hydrazine

**DOI:** 10.1107/S160053680801667X

**Published:** 2008-06-07

**Authors:** Hong-ai Li, Chang Su, Bao Li, Tie Chen, Bing-zhu Yin

**Affiliations:** a Key Laboratory of Organism Functional Factors of Changbai Mountain, Yanbian University, Ministry of Education, Yanji 133002, People’s Republic of China; b State Key Laboratory of Supramolecular Structure and Materials, College of Chemistry, Jilin University, Changchun 130012, People’s Republic of China

## Abstract

The title mol­ecule, C_6_H_4_N_2_S_4_, has a crystallographically imposed centre of symmetry located at the mid-point of the N—N single bond. The mol­ecule is essentially planar: the two five-membered rings form a dihedral angle of 0.17 (6)°. The crystal packing exhibits short inter­molecular S⋯S contacts of 3.549 (2) Å.

## Related literature

For general background, see: Yoshita *et al.* (1983 ▶); Moore *et al.*, (1998 ▶); Taniguchi *et al.* (2003 ▶). For useful properties of related compounds, see: Andreu *et al.*, (2004 ▶); Guerin *et al.* (2002 ▶). For the synthesis of the starting material, 2-methyl­thio-1,3-dithiol­ium iodide, see: Challenger *et al.* (1953 ▶).
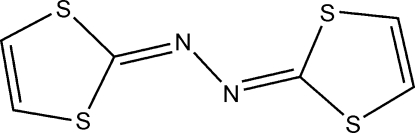


## Experimental

### 

#### Crystal data

C_6_H_4_N_2_S_4_
*M* *_r_* = 232.35
Monoclinic, 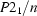

*a* = 3.9664 (3) Å
*b* = 10.122 (6) Å
*c* = 11.301 (8) Å
β = 97.39 (3)°
*V* = 449.9 (4) Å^3^
*Z* = 2
Mo *K*α radiation
μ = 0.99 mm^−1^
*T* = 291 (2) K
0.09 × 0.08 × 0.08 mm

#### Data collection

Rigaku R-AXIS RAPID diffractometer
Absorption correction: multi-scan (*ABSCOR*; Higashi, 1995 ▶) *T*_min_ = 0.919, *T*_max_ = 0.929
4256 measured reflections
1022 independent reflections
935 reflections with *I* > 2σ(*I*)
*R*_int_ = 0.021

#### Refinement

*R*[*F* ^2^ > 2σ(*F* ^2^)] = 0.024
*wR*(*F* ^2^) = 0.067
*S* = 1.07
1022 reflections
55 parameters
H-atom parameters constrained
Δρ_max_ = 0.46 e Å^−3^
Δρ_min_ = −0.18 e Å^−3^

### 

Data collection: *RAPID-AUTO* (Rigaku, 1998 ▶); cell refinement: *RAPID-AUTO*; data reduction: *CrystalStructure* (Rigaku/MSC, 2002 ▶); program(s) used to solve structure: *SHELXS97* (Sheldrick, 2008 ▶); program(s) used to refine structure: *SHELXL97* (Sheldrick, 2008 ▶); molecular graphics: *PLATON* (Spek, 2003 ▶); software used to prepare material for publication: *SHELXL97*.

## Supplementary Material

Crystal structure: contains datablocks global, I. DOI: 10.1107/S160053680801667X/cv2414sup1.cif

Structure factors: contains datablocks I. DOI: 10.1107/S160053680801667X/cv2414Isup2.hkl

Additional supplementary materials:  crystallographic information; 3D view; checkCIF report

## Figures and Tables

**Table 1:** Selected interatomic distance (Å)

S1⋯S2^i^	3.549 (2)

Symmetry code: (i) 
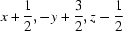
.

**Fig. 1.**
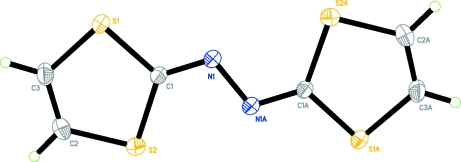
The molecular structure of (I) showing the atom numbering and 30% probalility displacement ellipsoids [symmetry code: (A) 2 - x, 1 - y, 1 - z].

## supplementary crystallographic information

### Comment

Within the field of molecular conductors, tetrathiafulvalene and its π-derivatives have played a leading role in the formation of organic charge-transfer complexes (Yoshita *et al.* 1983; Moore *et al.*, 1998; Taniguchi *et al.* 2003). One of the important strategies for the molecular design is to incorporate the nitrogen atom instead of carbon atoms to the conjugated spacer to modulate electron-donating properties (Andreu *et al.*, 2004; Guerin *et al.*, 2002). As a π-extended tetrathiafulvalene, we synthesized the title compound by incorporation an azino spacer between two 1,3-dithiole units.

The X-ray structure determination reveals that the title complex, (I) (Fig. 1), crystallizes in the monoclinic space group P2_1_/n space group.There is a half of the molecule in the asymmetric unit, and the inversion center lies on the mid-point of N—N bond. Two five-membered rings make a dihedral angle of 0.17 (6)°. In the absence of classical hydrogen bonds, the crystal packing exhibits short intermolecular S···S contacts of 3.549 (2) Å.

### Experimental

2-Methylthio-1,3-dithiolium iodide (Challenger *et al.*, 1953) (0.15 g, 0.54 mmol) and hydrazine monohydrate (0.13 g, 0.27 mmol) were dissovled in acetic acid (10 ml). The reaction mixture was refluxed for 2 h and then cooled to room temperature. The resulting solution was concentrated *in vacuo*. The yellow solid obtained was subjected to column chromatography (silica gel, dichloromethane) to afford the title compound as a pale yellow solid (0.08 g, 63.8% m.p. 481–483 K). Single crystals suitable for X-ray diffraction were prepared by slow evaporation of an acetone solution at room temperature.

### Refinement

H atoms were placed at calculated positions with C—H = 0.93 Å, and refined as riding, with *U*_iso_(H) = 1.2 *U*_eq_(C).

### Crystal data

**Table:**

C_6_H_4_N_2_S_4_	*F*_000_ = 236
*M**_r_* = 232.35	*D*_x_ = 1.715 Mg m^−^^3^
Monoclinic, *P*2_1_/*n*	Mo *K*α radiation λ = 0.71073 Å
Hall symbol: -P 2yn	Cell parameters from 3828 reflections
*a* = 3.9664 (3) Å	θ = 3.6–27.5º
*b* = 10.122 (6) Å	µ = 1.00 mm^−^^1^
*c* = 11.301 (8) Å	*T* = 291 (2) K
β = 97.39 (3)º	Block, yellow
*V* = 449.9 (4) Å^3^	0.09 × 0.08 × 0.08 mm
*Z* = 2	

### Data collection

**Table:**

Rigaku R-AXIS RAPID diffractometer	1022 independent reflections
Radiation source: fine-focus sealed tube	935 reflections with *I* > 2σ(*I*)
Monochromator: graphite	*R*_int_ = 0.021
*T* = 291(2) K	θ_max_ = 27.5º
ω scans	θ_min_ = 3.6º
Absorption correction: multi-scan(ABSCOR; Higashi, 1995)	*h* = −4→5
*T*_min_ = 0.919, *T*_max_ = 0.929	*k* = −13→13
4256 measured reflections	*l* = −14→14

### Refinement

**Table:**

Refinement on *F*^2^	Secondary atom site location: difference Fourier map
Least-squares matrix: full	Hydrogen site location: inferred from neighbouring sites
*R*[*F*^2^ > 2σ(*F*^2^)] = 0.024	H-atom parameters constrained
*wR*(*F*^2^) = 0.067	*w* = 1/[σ^2^(*F*_o_^2^) + (0.0401*P*)^2^ + 0.0828*P*] where *P* = (*F*_o_^2^ + 2*F*_c_^2^)/3
*S* = 1.07	(Δ/σ)_max_ < 0.001
1022 reflections	Δρ_max_ = 0.46 e Å^−^^3^
55 parameters	Δρ_min_ = −0.18 e Å^−^^3^
Primary atom site location: structure-invariant direct methods	Extinction correction: none

### Special details

**Table:**

Experimental. (See detailed section in the paper)
Geometry. All e.s.d.'s (except the e.s.d. in the dihedral angle between two l.s. planes) are estimated using the full covariance matrix. The cell e.s.d.'s are taken into account individually in the estimation of e.s.d.'s in distances, angles and torsion angles; correlations between e.s.d.'s in cell parameters are only used when they are defined by crystal symmetry. An approximate (isotropic) treatment of cell e.s.d.'s is used for estimating e.s.d.'s involving l.s. planes.
Refinement. Refinement of *F*^2^ against ALL reflections. The weighted *R*-factor *wR* and goodness of fit *S* are based on *F*^2^, conventional *R*-factors *R* are based on *F*, with *F* set to zero for negative *F*^2^. The threshold expression of *F*^2^ > σ(*F*^2^) is used only for calculating *R*-factors(gt) *etc*. and is not relevant to the choice of reflections for refinement. *R*-factors based on *F*^2^ are statistically about twice as large as those based on *F*, and *R*- factors based on ALL data will be even larger.

### Fractional atomic coordinates and isotropic or equivalent isotropic displacement parameters (Å^2^)

**Table:**

	*x*	*y*	*z*	*U*_iso_*/*U*_eq_	
C1	1.0199 (3)	0.62381 (14)	0.40299 (12)	0.0288 (3)	
C2	0.9210 (4)	0.87204 (16)	0.36541 (15)	0.0433 (4)	
H2	0.8668	0.9607	0.3732	0.052*	
C3	1.0268 (5)	0.82730 (15)	0.26661 (14)	0.0410 (4)	
H3	1.0495	0.8832	0.2028	0.049*	
N1	1.0475 (3)	0.50484 (12)	0.44228 (10)	0.0355 (3)	
S1	1.12148 (10)	0.66082 (4)	0.26068 (3)	0.03701 (14)	
S2	0.88557 (10)	0.76025 (4)	0.48070 (3)	0.03796 (14)	

### Atomic displacement parameters (Å^2^)

**Table:**

	*U*^11^	*U*^22^	*U*^33^	*U*^12^	*U*^13^	*U*^23^
C1	0.0330 (7)	0.0293 (6)	0.0243 (6)	−0.0017 (5)	0.0050 (5)	0.0016 (5)
C2	0.0545 (9)	0.0273 (7)	0.0482 (9)	0.0034 (7)	0.0067 (7)	0.0046 (7)
C3	0.0528 (9)	0.0313 (8)	0.0382 (8)	−0.0033 (6)	0.0040 (7)	0.0095 (6)
N1	0.0512 (8)	0.0299 (6)	0.0268 (6)	0.0005 (5)	0.0110 (5)	0.0014 (4)
S1	0.0519 (3)	0.0332 (2)	0.0278 (2)	−0.00120 (15)	0.01239 (16)	0.00342 (13)
S2	0.0487 (3)	0.0337 (2)	0.0333 (2)	0.00350 (15)	0.01252 (17)	−0.00133 (14)

### Geometric parameters (Å, °)

**Table:**

C1—N1	1.283 (2)	C2—H2	0.9300
C1—S1	1.7480 (17)	C3—S1	1.7296 (19)
C1—S2	1.7558 (16)	C3—H3	0.9300
C2—C3	1.322 (2)	N1—N1^i^	1.407 (2)
C2—S2	1.7448 (18)		
			
S1···S2^ii^	3.549 (2)		
			
N1—C1—S1	120.00 (11)	C2—C3—S1	117.41 (12)
N1—C1—S2	125.66 (11)	C2—C3—H3	121.3
S1—C1—S2	114.35 (8)	S1—C3—H3	121.3
C3—C2—S2	118.24 (13)	C1—N1—N1^i^	111.41 (14)
C3—C2—H2	120.9	C3—S1—C1	95.53 (8)
S2—C2—H2	120.9	C2—S2—C1	94.47 (9)

Symmetry codes: (i) −*x*+2, −*y*+1, −*z*+1; (ii) *x*+1/2, −*y*+3/2, *z*−1/2.
